# A Novel Approach to the Determination of Time- and Fatigue-Dependent Efficiency during Maximal Cycling Sprints

**DOI:** 10.3390/sports11020029

**Published:** 2023-01-28

**Authors:** Anna Katharina Dunst, Clemens Hesse, Olaf Ueberschär, Hans-Christer Holmberg

**Affiliations:** 1 Department of Endurance Sports, Institute for Applied Training Science, Marschnerstraße 29, 04109 Leipzig, Germany; 2 German Cycling Federation, 60528 Frankfurt am Main, Germany; 3 Department of Biomechanics, Institute for Applied Training Science, 04109 Leipzig, Germany; 4 Department of Engineering and Industrial Design, Magdeburg-Stendal University of Applied Sciences, 39114 Magdeburg, Germany; 5 Department of Physiology and Pharmacology, Biomedicum C5, Karolinska Institutet, 17177 Stockholm, Sweden; 6 Department of Health Sciences, Luleå University of Technology, 97754 Luleå, Sweden

**Keywords:** all-out exercise, efficiency, F/v profile, performance modelling, track cycling

## Abstract

Background: During maximal cycling sprints, efficiency (η) is determined by the fiber composition of the muscles activated and cadence-dependent power output. To date, due to methodological limitations, it has only been possible to calculate gross efficiency (i.e., the ratio of total mechanical to total metabolic work) in vivo without assessing the impact of cadence and changes during exercise. Eliminating the impact of cadence provides optimal efficiency (η_opt_), which can be modeled as a function of time. Here, we explain this concept, demonstrate its calculation, and compare the values obtained to actual data. Furthermore, we hypothesize that the time course of maximal power output (P_max_) reflects time-dependent changes in η_opt_. Methods: Twelve elite track cyclists performed four maximal sprints (3, 8, 12, 60 s) and a maximal-pedaling test on a cycle ergometer. Crank force and cadence were monitored continuously to determine fatigue-free force-velocity profiles (F/v) and fatigue-induced changes in P_max_. Respiratory gases were measured during and for 30 min post-exercise. Prior to and following each sprint, lactate in capillary blood was determined to calculate net blood lactate accumulation (ΔBLC). Lactic and alactic energy production were estimated from ΔBLC and the fast component of excess post-exercise oxygen consumption. Aerobic energy production was determined from oxygen uptake during exercise. Metabolic power (MP) was derived from total metabolic energy (W_TOT_). η_opt_ was calculated as P_max_ divided by MP. Temporal changes in P_max_, W_TOT,_ and η_opt_ were analyzed by non-linear regression. Results: All models showed excellent quality (R^2^ > 0.982) and allowed accurate recalculation of time-specific power output and gross efficiency (R^2^ > 0.986). The time-constant for P_max_(t) (τ_P_) was closely correlated with that of η_opt_ (τ_η_; r = 0.998, *p* < 0.001). Estimating efficiency using τ_P_ for τ_η_ led to a 0.88 ± 0.35% error. Conclusions: Although efficiency depends on pedal force and cadence, the latter influence can be eliminated by η_opt_(t) using a mono-exponential equation whose time constant can be estimated from P_max_(t).

## 1. Introduction

During cycling, the energy expended can be monitored by power meters that record the tangential force applied to the pedal or crank and the corresponding crank velocity during each revolution [1]. Power can be calculated as the product of the average force on the pedals and pedaling rate (e.g., [2,3,4]).

There is a specific relationship between the output of mechanical power (P) and the associated activation of the metabolic processes that provide the energy required in dynamic work lasting longer than one second [5]. The ratio between P and the corresponding energy expenditure is designated as efficiency (η) [6] and can be determined by synchronized determination of these two parameters [7].

Simultaneously, the energy supply can be estimated by employing indirect calorimetry based on the oxygen uptake and lactate accumulation in the blood before, during, and after exercise [5,7,8]. The energy consumed, the sum of the energy supply of dephosphorylation of PCr, glycolysis, and oxidative phosphorylation [5], is time-dependent, i.e., can be described as a function of time (ibid.).

To date, due to methodological limitations, it has only been possible to calculate gross efficiency (i.e., the ratio of total mechanical to total metabolic work) in vivo [9]. Thus, previous reports did not take changes in efficiency during (maximal) exercise into consideration, an important area for further investigation [10]. This is mainly due to the fact that no attempts have yet been made to describe the time-dependent energy supply or metabolic power, especially via anaerobic metabolic pathways, on the basis of real measured data [11]. However, in our recent study, we describe a novel approach for determining the kinetics of PCr dephosphorylation and accumulation of lactate in the blood based on data obtained during a 60-s maximal cycling sprint [12]. This approach might allow estimation of the time-dependent contribution of the phosphagenic and glycolytic pathways to energy production during exercise.

Muscular efficiency during cycling in vivo has been investigated at both maximal and submaximal intensities [6]. Sargeant [13] reported that this efficiency depends primarily on the mechanical efficiency of the fiber type(s) contributing to power output. In vitro, fibers containing myosin heavy chain (MHC) I demonstrate the greatest mechanical efficiency (as high as 35%), while MHC IIx fibers are the least efficient [14,15]. Efficiency tends to be higher when muscles shorten slowly as opposed to more rapidly (ibid.)

Factors that influence energy expenditure during exercise in vivo include the nature and intensity of the exercise, the muscle mass actively involved, and the coordination of movement required [7]. With respect to cycling, the highest η values (as great as 28%) are associated with low-intensity cycling [6,16,17,18], whereas in 30-s maximal cycling sprints, gross efficiency is less than 17% [19]. The lower efficiency in sprints reflects the more pronounced requirement for energy and higher pedaling rates, which lead to more extensive recruitment of MHC IIa and IIx fibers [7].

According to Henneman’s size principle [20], the energy demand determines a specific pattern by which muscles are recruited. During maximal efforts, all voluntary motor units are activated to supply the maximal energy required [21], but the actual power output is dependent on the pedaling rate [22]. Accordingly, muscular efficiency is determined both by the current pattern of recruitment and velocity-dependent power output [6].

Although the reliance of efficiency on pedaling rate during submaximal cycling has been demonstrated [6,18,23,24,25,26,27], in previous investigations on cycling efficiency, the effect of cadence on mechanical power and, thereby, efficiency was not normalized. Below, we will refer to the ratio of dynamic power output at any time during maximal exercise normalized to the cadence and the corresponding metabolic power associated with the greatest muscular efficiency as the optimal efficiency (η_opt_).

During maximal cycling sprints lasting as long as 60 s, P_max_(t) reflects fatigue-related changes in the F/v and P/v profiles [22,28], which might, in turn, reflect changes in the muscle fibers involved, with a successive reduction in the contribution of glycolytic, fast-twitch fibers. Assuming a dependency of η_opt_ on the recruitment patterns with an increasing efficiency with decreasing contribution of glycolytic muscle fibers [19,29], η_opt_(t) should be negatively correlated to P_max_(t).

The aims of the current investigation were as follows: (1) To calculate the optimal efficiency during maximal cycling sprints with normalization for and thereby elimination of the impact of cadence, (2) to monitor changes in this optimal efficiency as a function of time, and (3) to examine possible correlations between our model function describing optimal efficiency and the biomechanical model of maximal dynamic power output. Based on the discussion above, we predict that the classical calculation and our approach involving normalization for cadence will be in general agreement and, in addition, we hypothesize that the time courses of the optimal efficiency and maximal dynamic power output will be closely correlated.

More detailed insights into the time-dependent changes in efficiency during a maximal sprint and their relationship to the maximal available output of mechanical power at any given cadence would improve our understanding of metabolic and biomechanical response(s) to maximal effort, as well as potential interactions between these responses.

## 2. Methods

Since our investigation required high-level neuromuscular and metabolic performance, all 12 elite track cycling sprinters (three women: 18.7 ± 4.7 years, 177.0 ± 3.6 cm, 76.7 ± 4.6 kg; nine men: 22.2 ± 4.4 years, 184.3 ± 5.0 cm, 89.2 ± 6.0 kg; means ± standard deviations) who participated in this study had demonstrated essentially linear F/v profiles (R^2^ > 0.95) in previous tests and constant performance during all track cycling sprint races on a given day. They were requested to refrain from consuming alcohol and from intense training while maintaining their normal drinking habits and diet for 24 h prior to the experimental session. Before participating, all provided written informed consent, and the study was approved by the institute’s ethical committee (ER_2022.18.03_10) and performed in accordance with the Declaration of Helsinki. 

### 2.1. The Exercise Protocol

During a single visit to the laboratory, each participant performed four maximal sprints sitting on a cycle ergometer. Since this procedure was similar to the training performed and competition by these track cyclists, who had also carried out similar performance tests previously, no familiarization was considered necessary. All used their own cycling shoes and pedals, and the ergometer was set to place demands that resembled those encountered in actual competition.

Prior to each sprint, the participant warmed up with 6 min of low-intensity cycling (1–1.5 W kg^−1^ body weight), followed by a 3 s maximal sprint. Following 10 min of subsequent rest, each performed four maximal sprints lasting, successively, for 3, 8, 12, and 60 s in an isokinetic mode at a cadence of 120 rpm with a 1:2.4 deployment on an SRM cycle ergometer (Schoberer Rad Messtechnik GmbH, Jülich, Germany) with a 9-kg flywheel. With these settings, pedal force decreased linearly during the initial acceleration phase, and the target cadence of 120 rpm was reached within 4 s. In every test, the participant was asked to accelerate pedaling from approximately 20 to 120 rpm as rapidly as possible and to continue thereafter sprinting with maximal effort until the end, with loud verbal encouragement from the experimenters throughout.

Successive tests were separated by up to 2 h of rest, during each of which the athlete consumed a light meal containing the same amount of energy he/she was estimated to have expended during the preceding test. The next test started when the participant’s levels of blood lactate and blood glucose concentration (BGC) were comparable to those prior to the preceding test (BLC_pre_, BGC_pre_).

**Figure 1:**
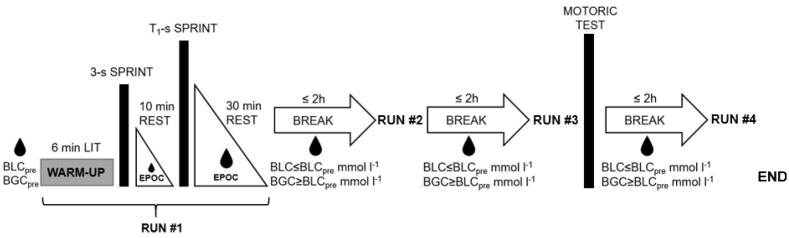
Schematic illustration of the study design. LIT: Cycling at low intensity; T_i_-s: Sprints where i = 1, 2, 3, 4 lasting for 3, 8, 12, and 60 s, respectively; EPOC: Measurement of the Excess Post-Exercise Oxygen Consumption during passive recovery; BLC_pre_: Pre-run blood lactate concentration; BGC_pre_: Pre-run blood glucose concentration; BLC: Blood lactate concentration; BGC: Blood glucose concentration. The drops depict time points or time spans when blood samples were taken.

The motoric test was carried out 30 min after the 12-s sprint and involved each participant cycling for 6 s at the maximal rate and with low resistance [30]. This setting enabled a pedaling rate of ≥160 rpm to be attained within the first 3 s, thereby generating data at high pedaling rates. The combination of these data with the fatigue-free rate of pedaling during the acceleration phase of each sprint allowed a more valid determination of the F/v profiles [31]. Figure 1 provides a schematic illustration of the entire exercise protocol.

Net crank torque (M) and angular velocity (ω) were monitored continuously at an internal sampling rate of 500 Hz utilizing an SRM power meter (Schoberer Radmesstechnik GmbH, Jülich, Germany). 

Each expired breath was analyzed continuously with a portable device (Metamax 3B, Cortex, Leipzig/Germany), starting 3 min before the beginning of the standardized warm-up (to determine resting oxygen uptake) until 30 min after the termination of exercise, during which period the participants sat still, moving as little as possible. 

Before and directly after each sprint, as well as 1, 3, 5, 7, 10, 15, 20, 25, and 30 min after the 8-, 12-, and 60-s sprints, 20 µL capillary blood was collected from the hyperemic ear lobe for hemolysis and enzymatic-amperometric determination of lactate (Biosen, EKF Diagnostics, Magdeburg, Germany). In the case of the 3-s test, accumulation of blood lactate was expected to be limited and, moreover, a considerable number of such samples had already been collected, so capillary blood was collected only after the first, third, fifth, and tenth minutes following this sprint. This ensured adequate blood flow, prevented sample contamination, and enhanced comfort.

### 2.2. Data Processing

Raw data are presented as power along with the corresponding pedaling rate, as sampled at 10 Hz. Since cadence was measured only once per revolution, the average tangential force F (N) on both pedals during a single revolution (taking into account the individual crank length), as well as the corresponding pedaling rate, were calculated from these values. 

### 2.3. External Power

To characterize time-dependent maximal external power output P, fatigue-free force-velocity and power-velocity profiles were created on the basis of the pedaling rate, and corresponding mean crank force during the first 3 or 4 revolutions (each lasting ≤3 s at a pedaling rate of 30–120 rpm) of the 60-s sprint or, in the case of the motoric test, during 1 or 2 revolutions at a pedaling rate exceeding 160 rpm [31]. Since pedaling rate is directly proportional to the tangential speed of motion at the pedal, movement velocity, and the corresponding mean crank force F were determined from this rate.

The F/v and P/v profiles were subjected to linear and non-linear regression analysis, respectively. The fatigue-free relationship between mean pedal force F and the movement velocity PR [32,33] is described by the function:

(1) F(v)=a·PR+b

P(v) is calculated by:

(2) $$P\left(v\right)=a\cdot {\mathrm{PR}}^{2}+b\cdot \mathrm{PR}$$

From these functions, maximal mean pedal force ($F_{\max}=b$), maximal pedaling rate (${\mathrm{PR}}_{\max}=-b/a$), maximal power output ($P_{\max}=-b^{2}/4a$) and the associated optimal pedaling rate (${\mathrm{PR}}_{\mathrm{opt}}=-b/2a)$ were obtained. All data were fitted by identifying the linear function with the highest coefficient of determination. Profile validity was considered to be valid if P_max_ ≥ P_peak_, the highest power output measured, and R^2^ ≥ 0.95.

To examine the loss in performance due to fatigue during the 60-s-sprint, the slope derived from the fatigue-free force-velocity profile (1) was maintained fixed, in accordance with Buttelli and colleagues [34]. As the F/v profile shifts, the characteristic parameters of the fatigue-free profiles also decrease [28] and, therefore, the fatigue-induced changes in the F/v are reflected in the change in a single parameter, such as the maximal power output (ibid.). Calculation of the instantaneous F/v profile and theoretical maximal power output for each data point i was based on the current cadence ${\mathrm{PR}}_{i}$ and corresponding mean crank force $F\left({\mathrm{PR}}_{i}\right)$:

(3) $$b_{i\;}=F\left({\mathrm{PR}}_{i}\right)-a\cdot {\mathrm{PR}}_{i}\;\Rightarrow P_{\max,i}{=-b}_{i}^{2}\cdot {\left(4a\right)}^{-1}$$

The decay in P_max_ during the 60-s sprint was approximated by applying non-linear regression analysis to a mono-exponential function of time t:

(4) $$P_{\max}\left(t\right){=A}_{P}\cdot e^{\frac{-t+\mathrm{TD}}{\tau_{P}}}{+C}_{P}$$

with $A_{P}{=P}_{\max}{(0)-C}_{P}$, where $C_{P}$ is the limiting value and τ_P_ the time constant of the function. In the case of Equation (4), a time delay (TD) was used to compensate for any potential delay in the onset of fatigue.

To avoid overestimation of the maximal power output in connection with the fatigue-free interval during the first seconds of exercise [30], P_max_(t) was there re-defined as follows:

(5) $$P_{\max}\left(t\right):=\{\begin{array}{c} P_{\max}\;\forall {\;t:\;A}_{P}\cdot e^{\frac{-t}{\tau_{P}}}{+C}_{P}\;\geq {\;P}_{\max}\\ A_{P}\cdot e^{\frac{-t}{\tau_{P}}}{+C}_{P}\;\mathrm{elsewhere}. \end{array}$$

### 2.4. Metabolic Power

To establish the phosphagenic energy supply during each sprint test, Excess Post-Exercise Oxygen Consumption ($\dot{V}O_{2\mathrm{EPOC}})$ was determined by non-linear regression using the following bi-exponential 4-parameter model [35]:

(6) $$\dot{V}O_{2\mathrm{EPOC}}\left(t\right){=A}_{\mathrm{FC}}\cdot e^{\frac{-t+\mathrm{TD}}{\tau_{\mathrm{FC}}}}{+A}_{\mathrm{SC}}\cdot e^{\frac{-t+\mathrm{TD}}{\tau_{\mathrm{SC}}}}{+\dot{V}O}_{2\mathrm{Base}}$$

where A_FC_ and A_SC_ represent the amplitudes of the fast and slow components, respectively, τ_FC_ and τ_SC_ the corresponding time constants, and $\dot{V}O_{2\mathrm{Base}}$ the asymptotic resting oxygen uptake as time approaches infinity. In the case of Equation (6), a time delay (TD) was used to compensate for any potential delay in measurement.

Replenishment of high-energy phosphates (VO_2PCr_, VO_2FC_) was estimated on the basis of the product of the amplitude A_FC_ and time constant τ_FC_ of the fast component (ibid.) The phosphagenic energy supply for each sprint was calculated as follows [8,36]:

(7) $$W_{\mathrm{PCr}}{=\mathrm{VO}}_{2\mathrm{PCr}}\cdot \mathrm{CE}\;$$

employing a caloric equivalent (CE) of 20.9 J mL^−1^ [8].

The time course of the change in blood concentration of lactate following each sprint was analyzed by non-linear regression, using a three-parameter model to determine the maximal concentration [37,38]:

(8) $$\mathrm{BLC}\left(t\right)=\frac{A\cdot k_{1}}{k_{1}{-k}_{2}}\cdot \left(e^{{-k}_{1}\cdot t}{-e}^{{-k}_{2}\cdot t}\right)+\mathrm{BLC}(0)$$

with A denoting the extra-vascular increase and k_1_ and k_2_ the rate constants for the accumulation and subsequent reduction in blood lactate, respectively. For each sprint test, the net accumulation of blood lactate ΔBLC (mmol L^−1^) was obtained by subtracting the pre-exercise level from the maximal level (BLC_max_-BLC(0)), as described by Mader (1994). The corresponding anaerobic lactic energy (W_BLC_) was calculated from ΔBLC in combination with body mass (BM [kg]) as follows:

(9) $$W_{\mathrm{BLC}}=\Delta \mathrm{BLC}\;\cdot {\mathrm{LAO}}_{2}E\;\cdot \mathrm{BM}\cdot \mathrm{CE}$$

When employing Equation (9), a caloric equivalent of 21.131 J mL^−1^, corresponding to a respiratory exchange ratio of >1.0, was used. Assuming that the distribution space of lactate is close to 50% of body mass, the O_2_-lactate equivalent (LAO_2_E) is 3.0 mL kg^−1^ mmol^−1^ [8]. 

Aerobic energy (W_AER_) was calculated from the increase in oxygen uptake during the 60-s sprint (VO_2_) and the caloric equivalent utilizing the following equation [36]:

(10) $$W_{\mathrm{AER}}{=\mathrm{VO}}_{2}\;\cdot \mathrm{CE}(\mathrm{RER})$$

In the case of Equation (10) CE is a function of the mean respiratory exchange ratio (RER = $\dot{V}{\mathrm{CO}}_{2}$ $\dot{V}O_{2}$^−1^) calculated as CE(RER) = 5.157 RER + 15.974 [J mL^−1^] for RER < 1 and set to 21.131 J mL^−1^ for RER ≥ 1 [8,36,39]. 

On the basis of the metabolic supply of energy associated with each sprint, the total metabolic cost ($W_{\mathrm{TOT}})$ was calculated as the sum of the contributions of phosphagenic, lactic, and aerobic energy:

(11) $$W_{\mathrm{TOT}}{=W}_{\mathrm{PCr}}{+W}_{\mathrm{BLC}}{+W}_{\mathrm{AER}}$$

The W_TOT_ associated with the individual 60-s sprint tests was employed to analyze the time course of the total metabolic supply of energy by non-linear regression utilizing the following mono-exponential 2-parameter model:

(12) $$W_{\mathrm{TOT}}\left(t\right){=A}_{w}\cdot {(1-e}^{\frac{-t}{\tau_{W}}})$$

where A_W_ represents the maximal supply of metabolic energy as time approaches infinity and τ_W_ the corresponding time constant. The time-dependent metabolic power MP(t) was calculated as the time derivative of function (12):

(13) $${\mathrm{MP}(t)=A}_{\mathrm{MP}}\cdot e^{\frac{-t}{\tau_{\mathrm{MP}}}}$$

where A_MP_ = A_W_ τ_W_^−1^ and τ_MP_ = τ_W_.

Optimal efficiency η_opt_ at each tenth of a second i during the 60-s sprint was determined as the ratio between time-dependent maximal P and MP:

(14) $$\eta_{\mathrm{opt},i}=\frac{P_{\max,i}}{{\mathrm{MP}}_{i}}\cdot 100$$

and this value applied to analyze the time course of optimal efficiency during the 60-s test by non-linear regression with the following mono-exponential three-parameter model:

(15) $$\eta_{\mathrm{opt}}\left(t\right)=H\cdot e^{\frac{t}{\tau_{\eta }}}{+H}_{0}$$

where H denotes the amplitude, H_0_ the baseline amplitude (i.e., efficiency at t = 0) and τ_η_ the time constant.

Gross efficiency at each tenth second i during the 60-s sprint was calculated as the ratio of external mechanical work (W_P_) to metabolic work:

(16) $$H_{i}=\frac{W_{P,i}}{W_{\mathrm{TOT},i}}$$

where W_P_ was either taken to be the sum of all external power values during the 60-s sprint (H_i_) or the sum of these same values recalculated according to Equation (2), with $b\left(t\right){=(-P}_{\max}\left(t\right)\cdot {4a)}^{0.5}$:

(17) $$P\left(v,t\right)=a\cdot {\mathrm{PR}}^{2}+b(t)\cdot \mathrm{PR}$$

### 2.5. Statistical Analyses

The distribution of all data sets was confirmed to be normal using the Shapiro–Wilk test, and values are presented as means ± SD. The measured and modeled data were compared by linear regression, and the relative difference was employed to determine the bias of the measurements. The mean differences between values for the male and female participants were compared using t-tests for independent samples. 

The Pearson product-moment correlation test was used to assess interrelationships between variables and the resulting correlation coefficient r (small: r ≥ 0.1; medium: r ≥ 0.3; large: r ≥ 0.5), together with Cohen’s d (small: d ≥ 0.2; medium: d ≥ 0.5; large: d ≥ 0.8), employed as a measure of effect size. In connection with the regression analysis the least squares procedure was applied to determine the parameters of the various model functions. The quality of the regression analyses was examined by calculating the coefficient of determination R^2^. 

An alpha level of <0.05 was considered to be statistically significant. All mathematical analyses and statistical tests were carried out using the IBM SPSS statistics Software for Windows (version 24, SPSS Inc., Chicago, IL, USA), Office Excel 2016 (Microsoft Corporation, Redmond, WA), and MATLAB 9.10.0 R2021a (The MathWorks, Inc., Natick, MA, USA).

## 3. Results

### 3.1. External Power

**Figure 2:**
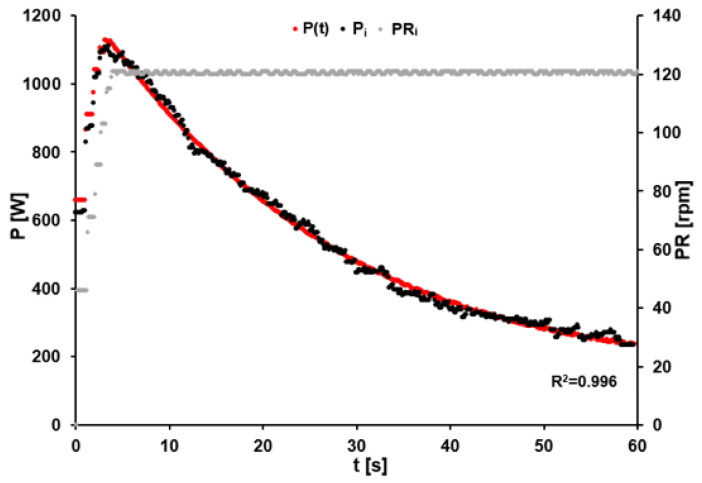
Power output (black dots) and pedaling rate (grey dots) of an elite track cyclist during the isokinetic (120 rpm) 60-s all-out sprint on an SRM-ergometer (sampling rate of 10 Hz) with a mean power output of 551 W and power output prediction (red dots) calculated based on the time-dependent maximal power output and the current pedaling rate using Formula (17).

After an acceleration phase of less than 4 s, all athletes reached the target cadence of 120 rpm during the 60-s sprint. The participants achieved a peak power output of 1460 ± 241 W after 3.74 ± 1.04 s and completed the effort with an average power output of 677 ± 115 W. Figure 2 illustrates the time course of power output and corresponding pedaling rate for one athlete.

**Figure 3:**
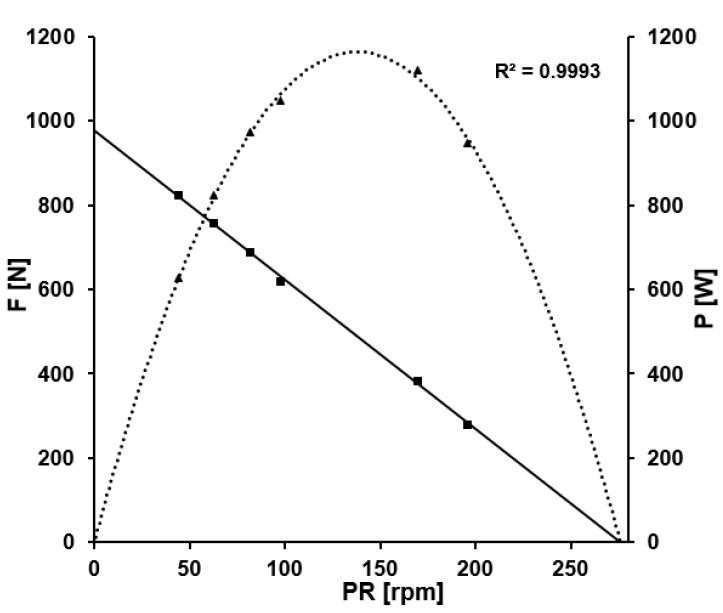
The fatigue-free force-velocity and power-velocity profiles of a track cycling sprinter. The black line represents the fatigue-free relation of mean pedal force and pedaling rate (derived from the data points represented by the black squares), the black dotted curve depicts the resulting power output, the black triangles the associated data points. Maximal mean pedal force was 960 N, maximal pedaling rate was 282 rpm leading to a fatigue-free optimal pedaling rate of 141 rpm.

With respect to the fatigue-free F/v profile associated with this same 60-s effort, the mean maximal force was 1210 ± 196 N, mean maximal crank velocity 300 ± 24.3 rpm and optimal pedaling rate 150 ± 12.2 rpm. The slope of this profile was a=-4.04 ± 0.72 N rpm^−1^ and the R^2^ ≥ 0.99 for all of the athletes. Figure 3 shows the fatigue-free force-velocity and power-velocity profiles for the same athlete whose data are depicted in Figure 2.

**Figure 4:**
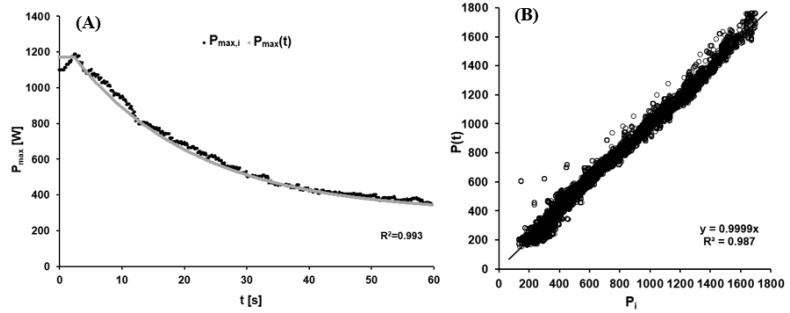
(**A**) The dynamic maximal power output as a function of time reflecting the increase in fatigue with prolonged cycling. Black points: Current maximal power output calculated for data point i (P_max,i_) using Equation (3). Grey curve: Time-dependent maximal power output (P_max_(t)) calculated using Formula (5) with A_P_ = 989 W, τ_P_ = 19.69 s, C_P_ = 294 W, and TD = 2.6 s. (**B**) Comparison of measured (P_i_) and modeled data (P(t)) for the cadence-dependent power output via linear regression analysis.

The values of the mean model function $P_{\max}\left(t\right)$ were $A_{P}=1501.09\;\pm \;357.82$ W, $\tau_{P}=20.12\;\pm \;4.19$ s and C=297.72 ± 75.87 W. The high quality of the non-linear regression was indicated by its R^2^ of 0.990 ± 0.005. As a test of the model, Figure 4A depicts a representative fitting of the function describing maximal power output to the actual data collected, and Figure 4B the difference between the modeled and measured data for all twelve athletes. The bias of 2.50 W with a standard deviation of 45.5 W indicated satisfactory overall agreement.

### 3.2. Metabolic Power

The mean parameters employed to model EPOC(t) were A_FC_ = 2.49 ± 0.62 L min^−1^, τ_FC_ = 0.84 ± 0.13 min, A_SC_ = 0.51 ± 0.34 L min^−1^, τ_SC_ = 7.34 ± 3.67 min and $\dot{V}O_{2\mathrm{Base}}=0.42\;\pm \;0.09{\;L\;\min}^{-1}$. The average R^2^ of all of these models was 0.895 ± 0.047. The mean net oxygen uptake during the fast component of EPOC, mean net accumulation of blood lactate ΔBLC, and mean oxygen uptake during exercise are documented in Table 1.

**Figure 5:**
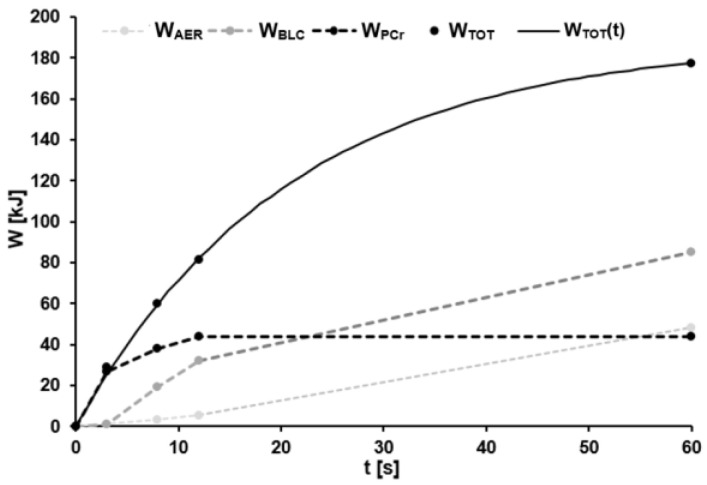
Total energy supply (W_TOT_) in the 60-s effort based on the sum of the energy contributions from the phosphagenic (W_PCr_), anaerobic lactic (W_BLC_), and aerobic (W_AER_) energy pathway during each effort. Parameters of the model function calculated with Formula (12) were A_W_ = 188 kJ, τ_W_ = 20.85 s. The coefficient of determination was R^2^ > 0.99.

In the case of the time-dependent supply of total metabolic energy, the mean parameters were A_W_ = 233.38 ± 31.84 kJ and τ_W_ = 21.31 ± 2.73 s with R^2^ ≥ 0.99. Derivation MP(t) of the model function W_TOT_(t) gave values of A_MP_ = 11262.66 ± 1743.40 W and τ_MP_ = τ_W_. Figure 5 illustrates a representative example of the estimated energy supplied by the three different pathways, as well as the sum of these three during the 60-s sprint.

### 3.3. Optimal Efficiency and Recalculation of Gross Efficiency

**Figure 6:**
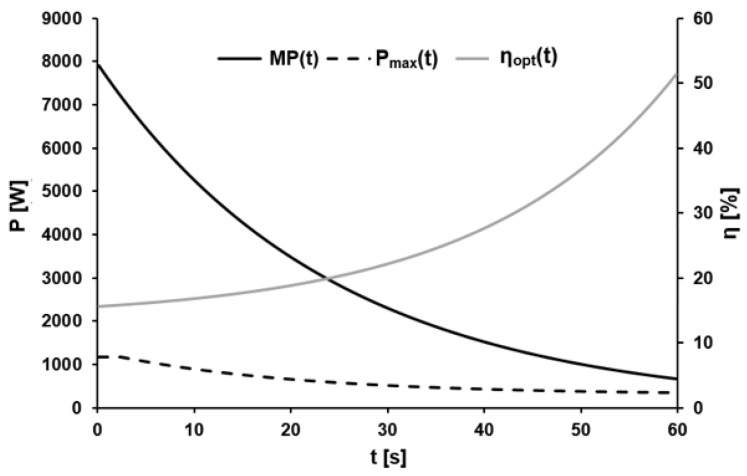
Exemplary metabolic power MP(t), maximal external power P_max_(t), and the optimal efficiency η_opt_(t) over the course of time.

The mean parameters associated with the dynamic optimal efficiency were H = 2.47 ± 1.38%, τ_η_ = 20.38 ± 4.18 s, and H_0_ = 13.42 ± 2.39% with R^2^ ≥ 0.99. Figure 6 shows a representative example of the time course of the calculated metabolic and maximal external power, as well as the ratio between these two parameters, i.e., the optimal efficiency during the 60-s sprint.

**Figure 7:**
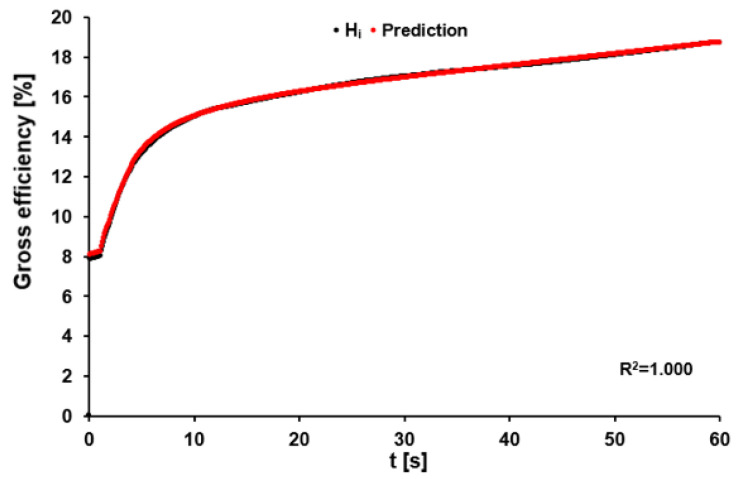
Gross efficiency calculated for data point i (H_i_) using Equation (16) (black dots), and predicted time-dependent gross efficiency calculated based on the sum of recalculated P-data using Formula (17).

Gross efficiency during the 60-s sprint increased continuously from 10.01 ± 2.18% at 3 s, over 13.51 ± 1.91% at 8 s, and 14.55 ± 1.79% at 12 s to 18.42 ± 1.51% after 60 s. Testing of the model for accurate prediction of the time-specific gross efficiency using Formula (16) resulted in a bias of 0.18 ± 0.32% with R^2^ = 0.995 ± 0.003. A representative example is shown in Figure 7.

### 3.4. Correlation Analyses

**Figure 8:**
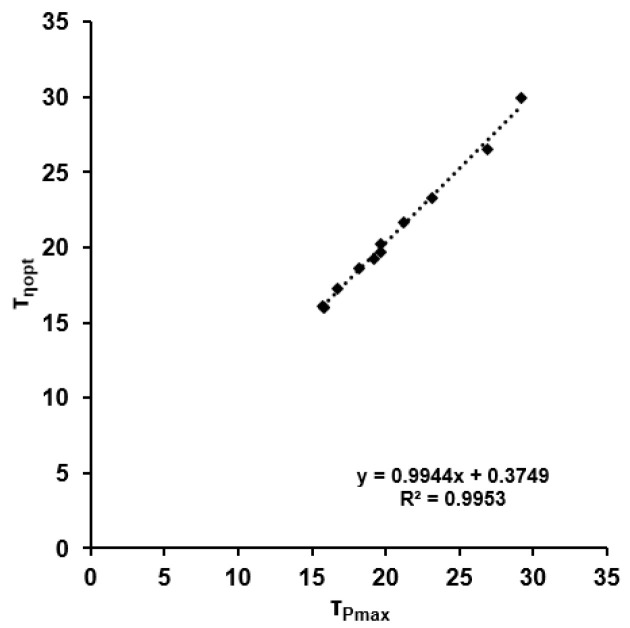
Linear relationship between the time constants of dynamic maximal power output and optimal dynamic efficiency.

τ_MP_ was highly correlated with τ_η_ (r = 0.998, *p* < 0.001), but there was no correlation between τ_WTOT_ and τ_MP_ or τ_η_ (r < 0.541; *p* > 0.5). The results of the linear regression are shown in Figure 8. Estimation of the efficiency employing τ_P_ for τ_η_ resulted in a mean error of 0.88 ± 0.35%.

Although the maximal mean pedal force (*p* < 0.001, d = 3.743), maximal pedaling rate (*p* < 0.028, d = 1.822), maximal fatigue-free power output (*p* < 0.004, d = 4.965), and the amplitudes A_P_ (*p* < 0.003, d = 2.864) and A_W_ (*p* < 0.001, d = 2.573) of the female cyclists were significantly smaller than those of the men, who also attained greater mean power output than the women during the 60-s sprint (*p* < 0.001, d = 4.202), there were no significant sex differences with respect to model quality (*p* > 0.9, d < 0.06) or the correlation between τ_P_ and τ_η_ (r ≥ 0.998, *p* < 0.001).

## 4. Discussion

The major finding of the present investigation is that optimal efficiency, i.e., the ratio of the time-dependent maximal power output and corresponding metabolic power during a 60-s maximal sprint by elite track cyclists can be determined with sufficient accuracy using a mono-exponential equation. The value obtained for this optimal relationship is usually distorted by the difference between the actual and optimal pedaling rate at any given time point. Combining the fatigue-induced changes in the F/v profile with the current cadence allows for modeling of the actual values of power output and gross efficiency in a consistent manner with previous observations. Our current findings confirm that the reduction in maximal power output during a 60-s maximal sprint is accompanied by a fatigue-induced incline in the optimal efficiency, presumably reflecting a change in the composition of the muscle fibers activated.

All of the mathematical models described here demonstrate excellent quality, making them suitable for describing the time-dependent behavior of the total energy supply (W_TOT_) and dynamic maximal power output (P_max_(t)) during an isokinetic 60-s all-out test on a cycle ergometer.

### 4.1. Metabolic Power

The mono-exponential function describing W_TOT_(t) exhibits a relatively rapid increase in energy supply during the first seconds of maximal exercise, with the half-maximal value of this parameter being attained after ~15 s. The values for metabolic work of ~175 kJ after 30 s and ~219 kJ after 60 s of maximal sprinting obtained here are in line with several earlier reports on energy supply during maximal 30- to 60-s sprints (Wingate Test) (e.g., [19,40,41,42,43,44,45]). Since the peak and mean power outputs observed here (approximately 1500 W and 700 W, respectively) exceed those reported in these earlier publications, our somewhat higher values for W_TOT_(t) were to be expected. Our higher values for metabolic work reflect primarily a more extensive accumulation of lactate in the blood and greater mean oxygen uptake, which is not surprising for our group of elite athletes. Our model for W_TOT_(t) provides values that are highly similar to the actual measurements.

### 4.2. External Power

As is the case for W_TOT_(t), P_max_(t) can be described accurately by a mono-exponential function which confirmed the performance of maximal effort by our cyclists during the 60-s sprint. The observation that the application of the actual cadence to the function (Formula (17)) provides a very good prediction of the non-normalized gross efficiency further establishes the validity of this equation.

The finding that none of our participants attained the maximal calculated power output during the sprints is in line with our previous observations [28,30]. Due to the initial resistance during the acceleration phase of the sprints, when the athletes reached the point of producing the highest power, they were potentially fatigued. Accordingly, our results show that the participants had already accumulated significant levels of lactate in their blood prior to reaching P_Peak_ and were already fatigued at this point, applying the time to peak to P_max_(t).

After sprinting for ~3 s, maximal power output decreased exponentially with time, with a time constant of ~20 s, reaching its half-maximal value after ~14 s. Towards the end of each sprint, the maximal power approached a plateau of ~300 W. 

### 4.3. Optimal Efficiency

The dynamic maximal power output P_max_(t) divided by the derivative of the metabolic energy function (metabolic power function) MP(t) was utilized to determine the parameters of the model of cadence-normalized optimal efficiency η_opt_(t). This revealed that η_opt_(t) can be described with sufficient accuracy by a mono-exponential function that changes with time in a direction opposite to that of P_max_(t). The increase in optimal efficiency from ~16% during the first second to ~24% after 30 s and then to ~58% after 60 s gave a time constant of ~20 s, in very good agreement with the time constant for P_max_(t). 

At the beginning of each sprint, all muscle fibers that can be recruited voluntarily are activated, leading to extensive phosphagenic flux and successive activation of the glycolytic and aerobic fluxes. With the onset of fatigue after ~3 s, the phosphagenic flux decreases significantly, while the glycolytic flux is activated maximally and the oxidative flux remains only partially activated [12]. Between 12 and 60 s of cycling, the phosphagenic flux reaches a plateau, with the glycolytic flux continuing to contribute and the oxidative flux contributing progressively to total energy production (ibid.). The declining anaerobic flux is associated with elevated efficiency and a reduction in maximal power output, indicating that the contribution of predominantly glycolytic fibers lessens [19]. Assuming that the time course of P_max_(t) reflects the time course of the recruitment of the different types of fibers in the propulsive muscles, as well as their metabolic state, this finding is reasonable.

The limits of optimal efficiency calculated here far exceed such values reported in the literature. In our case, optimal efficiency rose exponentially from ~16% when fatigue-free to ~58% at the end of the sprint. Although the maxima calculated are unexpectedly high, recalculation as gross efficiency, which ranged from ~10% 3 s after the start to ~18% at the end of the sprint, gave values that are in close agreement with comparable published values. For example, the literature values correspond well to our current findings interpolated for 30 s of load [7,19]. One major cause for our high values for optimal efficiency appears to be our normalization with respect to cadence. However, despite the overall reasonableness of our results, we cannot fully explain why our maxima for η_opt_(t) exceed the upper limits of muscular efficiency reported previously, both in vivo and in vitro.

## 5. Practical Applications

The close agreement between the time constants for optimal efficiency and maximal power output observed here enables prediction of the time course of efficiency during maximal cycling sprints based on continuous measurement of power. This novel approach allows reconstruction of metabolic status during exercise on the basis of a single sprint test.

## 6. Limitations

Our study involves considerable mathematical modeling of physiological responses during maximal exercise. Despite the high quality of our individual models, it must be remembered that this approach simplifies interrelationships and systematic behavior. 

The models employed here are based on data derived from several maximal sprint tests. Therefore, their validity requires actual maximal performance by the athletes and reliable determination of parameters in the laboratory. Despite our efforts to standardize the tests, the metabolic response during the 60-s effort has been reconstructed indirectly, so misinterpretation cannot be ruled out entirely. 

Determination of the propulsive work performed on the cycle ergometer alone here, ignoring losses due to non-tangential forces on the crank, could lead to an underestimation of the total mechanical work. At the same time, the metabolic costs of non-tangential forces, accompanying movements, and stabilization of body parts are included in the metabolic expenditure. Both of these parameters could have led to an underestimation of the actual efficiency, the validity of which must therefore be assessed critically. Furthermore, our two-compartment model is highly simplified, and other factors might have influenced efficiency as well. However, at present, there is no reliable procedure for quantifying efficiency in vivo.

Due to the limited number of our participants, their elite training status and the other potential sources of error considered above, confirmation of our findings is required, in particular utilizing different cohorts. When recruiting participants for such an investigation, care must be taken to ensure adequate capability and proficiency to sustain maximal neuromuscular performance. If performance is below the time-dependent maximum, metabolism in the fibers of the active muscles may vary, altering the overall metabolic response.

## 7. Conclusions

Both efficiency and power output depend on pedal force and pedaling rate. At the same time, calculating the time-dependent optimal pedaling rate as the ratio between time-dependent maximal power output and the corresponding metabolic power eliminates the impact of pedaling rate on efficiency. Due to fatigue, maximal power output decreases exponentially with time, whereas efficiency increases with an almost identical time course. The close agreement between the time course of optimal efficiency and dynamic maximal power output enables a novel approach to the estimation of the time-dependent metabolic costs based on biomechanical measurements.

## Figures and Tables

**Table 1:** Mean oxygen uptake of the fast component of EPOC, mean net blood lactate accumulation and mean oxygen uptake during each sprint (3, 8, 12, 60 s; N = 12).

Parameters	3-s	8-s	12-s	60-s
VO_2FC_ [mL]	1441 ± 349	2039 ± 462	2256 ± 473	2550 ± 572
ΔBLC [mmol L^−1^]	0.64 ± 0.24	4.58 ± 0.66	7.98 ± 0.89	17.69 ± 2.11
VO_2_ [mL]	75 ± 23	226 ± 57	386 ± 98	3220 ± 536

Abbreviations: VO_2FC_: Oxygen uptake of the fast component of EPOC, ΔBLC: Net blood lactate accumulation, VO_2_: Oxygen uptake during exercise.

## Data Availability

The datasets generated and/or analysed in connection with the present investigation can be obtained from the corresponding author upon reasonable request.

## Abbreviations

a	Slope of the fatigue-free F/v profile
A	Amplitude of the extra-vascular lactate concentration
A_FC_	Amplitude of oxygen consumption during the fast component of EPOC
A_MP_	Amplitude of the metabolic power
A_P_	Amplitude of the maximal dynamic power output
A_SC_	Amplitude of oxygen consumption during the slow component of EPOC
A_w_	Amplitude of the metabolic work
b, b_i_	the intercept of the F/v profile with the *y*-axis for data point i
BGC	Blood glucose concentration
BLC	Blood lactate concentration
${\mathrm{BLC}}_{\max}$	Maximal blood lactate concentration
BLC(t)	Post-exercise blood lactate concentration
BLC(0)	Pre-exercise blood lactate concentration
BM	Body mass
CE	Caloric equivalent
C_P_	The limiting value of maximal dynamic power output
EPOC	Excess Post-Exercise Oxygen Consumption
F	Mean pedal force
F/v	Force/velocity
F(v)	Mean pedal force as a function of pedaling rate
F(PR_i_)	Mean pedal force in connection with cadence i
H	The amplitude of optimal efficiency
H_i_	Gross efficiency associated with data point i
H_0_	The baseline amplitude of optimal efficiency
	The ith segment of the total time subdivided into n segments
k_1_	Rate constants for the appearance of BLC
k_2_	Rate constants for the disappearance of BLC
LAO_2_E	$O_{2}$–lactate equivalent
MHC	Myosin heavy chain
MP	Metabolic power
P	Output of external/mechanical propulsive power
P/v	Power/velocity
P(v)	Mechanical power output as a function of pedalling rate
PCr	Phosphocreatine
P_max_, P_max,i_	Maximal power output derived from P/v profile, maximal power output for data point i
P_max_(t)	Maximal mechanical power output as a function of time, i.e., the dynamic maximal power output
PR, PR_i_	Pedaling rate, cadence associated with data point i
PR_max_	Maximal pedaling rate, maximal cadence
RER	Respiratory exchange rate
rpm	Revolutions of the crank per minute, cadence
T	Exercise duration
TD	Time delay
v	Velocity of the crank or pedal
$\dot{V}O_{2}$	Oxygen uptake
VO_2_	Oxygen consumption during exerise
$\dot{V}O_{2\mathrm{Base}}$	Baseline of oxygen uptake at rest
$\dot{V}O_{2\mathrm{EPOC}}$	Excess Post-Exercise Oxygen Consumption
${\mathrm{VO}}_{2\mathrm{PCr}}$	Oxygen consumption required to replenish PCr post-exercise; oxygen consumption during the fast component of EPOC
VO_2SC_	Oxygen consumption during the slow component of EPOC
W_AER_	Aerobic energy supply
W_BLC_	Lactic energy supply
W_P_, W_P,i_	External work, external work for data point i
W_PCr_	Phosphagenic energy supply, alactic energy supply
W_TOT_, W_TOT,i_	Total energy supply, total energy expended during pedal stroke i
ΔBLC	Blood lactate accumulation
η	Efficiency
η_opt_,_i_	Optimal efficiency for data point i
η_opt_	Optimal efficiency
τ_FC_	Time constant of the fast component of EPOC(t)
τ_SC_	Time constant of the slow component of EPOC(t)
τ_P_	Time constant of maximal external power output
τ_MP_	Time constant of metabolic power
τ_W_	Time constant of metabolic work
τ_η_	Time constant of optimal efficiency
∈	In
∀	For all
